# Levels, Sources and Toxicity Risks of Polycyclic Aromatic Hydrocarbons at an Island Site in the Gulf of Tonkin

**DOI:** 10.3390/ijerph17041338

**Published:** 2020-02-19

**Authors:** Xiaoyang Yang, Shijie Liu, Yuanguan Gao, Wenjuan Zhao, Yu Liu, Jingying Mao, Zhaoyu Mo

**Affiliations:** 1 State Key Laboratory of Environmental Criteria and Risk Assessment, Chinese Research Academy of Environmental Sciences, Beijing 100012, China; yangxy@craes.org.cn (X.Y.); gaoyg@craes.org.cn (Y.G.); zhaowj@craes.org.cn (W.Z.); 2 School of Pharmacy, Liaoning University, Shenyang 110036, China; liuyu0710@163.com; 3 Scientific Research Academy of Guangxi Environmental Protection, Nanning 530022, China; 13978185061@163.com (J.M.); 18878738561@163.com (Z.M.)

**Keywords:** PAHs, Gulf of Tonkin, diagnostic ratio, back trajectory analysis, toxicity risk

## Abstract

The varying concentrations of polycyclic aromatic hydrocarbons (PAHs) at remote islands is an important indicator, demonstrating the contributions from different regional combustion sources. In this study, gaseous and particulate PAHs were measured at Weizhou Island in the Gulf of Tonkin from 15th March to 14th April, 2015. The concentrations of PAHs ranged from 116.22 to 186.74 ng/m^3^ and from 40.19 to 61.86 ng/m^3^ in gas and particulate phase, respectively, which were much higher than those of some remote sites in Asia. Phenanthrene, fluoranthene, pyrene, and chrysene, which were mainly found in diesel vehicle emissions, had relatively high concentrations in both gas and particulate phases. According to the comprehensive results of back trajectory cluster analysis and diagnostic ratios, the local vessel emission was probably the main source of PAHs, which was much more important than the coal and biomass combustion sources from remoter regions. The toxicities represented by ∑PAH_7_, benzo(*a*)pyrene-equivalent carcinogenic power, and 2,3,7,8-tetrachlorodibenzo-p-dioxin-based total toxicity potency are much higher in particulate phase than those in gas phase. However, the toxicities of gas phase should not be neglected from the point of view of indirect-acting mutagenicities due to the high contribution of fluoranthene.

## 1. Introduction

Much attention is being paid to the presence of polycyclic aromatic hydrocarbons (PAHs), which are carcinogenic and/or mutagenic chemicals, in the environment [1,2]. PAHs in the atmosphere mainly originate from the incomplete combustion of fossil fuels and biomass, such as motor-vehicle emission, heating supply, energy production and crop residue burning [3,4,5,6,7]. PAHs have been found in both the particulate and gaseous phases of combustion emissions and the ambient atmosphere due to their semi-volatile characteristics [8,9,10]. Gas-particle partitioning is also an important factor influencing the fate of PAHs in the environment, such as long-range atmospheric transport, transformation between phases, and removal from the atmosphere via wet and dry deposition [11,12].

**Figure 1:**
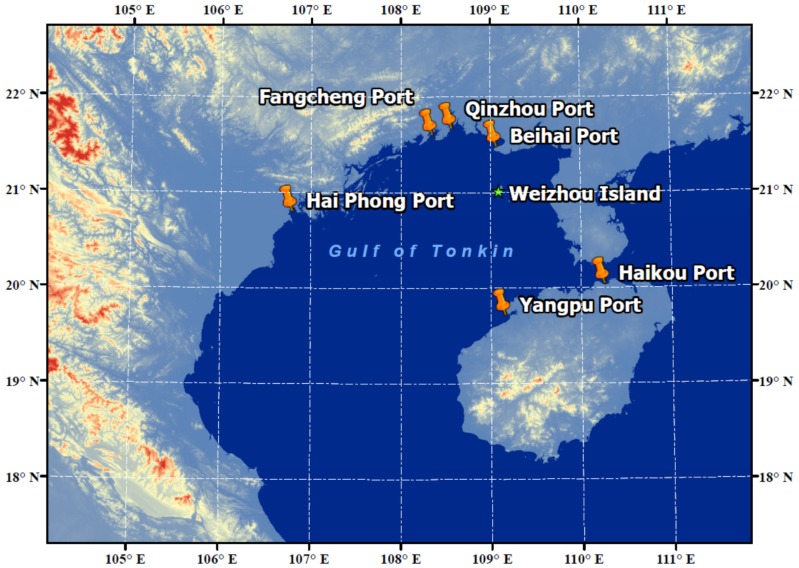
Location of the Weizhou Island in Gulf of Tonkin (GT).

The Gulf of Tonkin (GT) is located in the northwest of South China Sea, half-surrounded by southern China mainland, Hainan Island, and Indochina Peninsula. The two Chinese provinces connected to GT, Guangxi, and Guangdong, contributed around 13.07% of the total GDP in China in 2018 [13]. Economic activity has a close connection to the emission of pollutants, and a lot of relative studies concerning the issue of air pollution in these two provinces have been reported [14,15,16,17]. Moreover, springtime emissions from biomass burning in the Indochina Peninsula often cause serious air pollution and can sometimes influence the air quality in the downwind regions by long-range transport [17,18,19,20,21]. Additionally, there are 5 Chinese ports (Yangpu, Haikou, Fangcheng, Beihai and Qinzhou) as well as 1 Vietnamese port (Hai Phong) in the surrounding area of GT (Figure 1). The total handling capacity of the aforementioned 6 ports was 407.31 million tons in 2017 [22]. The influences of the emissions from ships and ports on the atmosphere over GT should not be neglected either. It is of great interest to clarify the levels, contributors, and toxicity risks of PAHs in GT, and improve our understanding of PAH pollution in a marine area surrounded by various emission sources.

Remote islands are often treated as background sites for studies on the regional atmospheric environment by comprehensive analysis of observation and/or simulation data [23,24,25]. The insular PAHs are also well-investigated to clarify the origin of the polluted air masses by deeply analyzing various PAH species [26,27]. A considerable amount of research has been carried out based on the atmospheric observations of some remote islands in the Yellow Sea or the East Sea focusing on the topic of regional air pollution in North East Asia [28,29,30].

In this study, gaseous and particulate PAHs were measured at Weizhou Island in GT from 15th March to 14th April 2015. Back trajectories during the sampling period and diagnostic ratios of PAHs were analyzed, in order to find out the contributions of different possible emission sources at this site in spring. Finally, the toxicity risk of PAHs was also evaluated.

## 2. Materials and Methods

### 2.1. Sample Collection

Particulate- and gas-bound PAHs were collected simultaneously by a medium-volume air sampler at a flow rate of 78 L/min from 15th March to 14th April, 2015 at Weizhou Island (21.01° N, 109.10° E), located in GT, China (Figure 1). This small island is 40 km south to Beihai, Guangxi Province and 60 km east to the Leizhou Peninsular, Guangdong Province. The sampler was set on the roof of a 15 m high building. The pre-combusted (450 °C for 6 h) quartz fiber filters (90 mm, Munktell) were used for the collection of particulate-bound PAHs. The gaseous PAHs were collected on XAD-4 resin packed in a column with polyurethane foam at each end. The column was connected directly under the filter described above. The XAD-4 resin (diameter 250–800 µm, pore size 48 Å, volume/weight ratio 0.96 cm^3^/g, surface area 725 m^2^/g) was purchased from Rohm and Haas (Philadelphia, PA, USA). Filters and resin-packed columns were daily changed at 7 a.m., and 26 sets of samples were obtained in total. After sampling, the filters and resin columns were both resealed and stored at –20 °C in darkness until analysis. Field blank samples were taken by setting the filter and resin column to the sampler for a few seconds without pumping.

### 2.2. Chemicals

The 18 PAHs listed in Table 1 were analyzed. PAH standards as well as the internal standards pyrene (Pyr)_-*d10*_ and benzo[*a*]pyrene_-*d12*_ were purchased from ChemService (West Chester, PA, USA). All chemicals used were of analytical grade.

### 2.3. Chemical Analysis

With the internal standards added, filter and resin samples were extracted three times with 80 mL of a 3:1 *v/v* mixture of benzene and ethanol (15 min each time) and three times with 640 mL of acetone (30 min each time), respectively, in an ultrasonic extraction instrument. The extract was evaporated to dryness, then part was re-dissolved in acetonitrile and PAHs in the extract were analyzed by Gas Chromatography-Mass Spectrometer (GC-MS).

Qualitative and quantitative GC-MS analyses of PAHs were carried out with an Agilent 7890A GC system equipped with an HP-5 column (30 m × 0.25 mm × 0.25 μm), coupled to an Agilent 5975 MS Engine mass spectrometer (Agilent Technologies, Santa Clara, CA, USA). Chromatographic conditions were as follows: injector temperature 50 °C; thermal program: 75 °C for 1 min, ramping 25 °C/min up to 150 °C, 4 °C/min up to 235 °C, 3 °C/min up to 265 °C, 50 °C/min up to 300 °C for 11.94 min; carrier gas helium, 1 mL/min, injection volume 1 μL, temperature transfer line 300 °C. Electronic ionization at 70 eV, scanning: full scan-mass range 98–310 m/z. The chromatogram was obtained in the total ion mode, and the molecular ion of each PAH and internal standards were then extracted.

The recovery for the extraction and clean up procedure was 97.3 ± 5.2% (mean and standard deviation for all compounds). The limit of detection and limit of quantitation were estimated in pg/m^3^ and ranged from 0.003 to 0.054 for PAHs.

### 2.4. Back Trajectory Cluster Analysis

Back trajectory analysis was used to provide information on air mass origins over the sampling period and investigate the sources of PAHs at Weizhou Island. Back trajectories were calculated using the Hybrid Single-Particle Lagrangian Integrated Trajectory (HYSPLIT) model developed by the National Oceanic and Atmospheric Administration (NOAA). The monthly Global Data Assimilation System (GDAS, global, 2006-present) dataset was obtained from the National Centers for Environmental Prediction (NCEP) for the model calculation. In this study, 3-day backward air trajectories were generated at 3 h intervals from 15th March to 14th April arriving at the height of 500 m above the ground level at Weizhou Island.

In order to distinguish different source regions, cluster analysis of back trajectories was used to group similar air mass origins together. By grouping similar trajectories, the information on pollutant with similar chemical histories can be obtained from the post-processed data. The key issue in cluster analysis is how to describe the similarity of different clusters, which is usually indicated by distance matrix. In the HYSPLIT model, there are two kinds of distance measurements, namely Euclidean distance, and angle-based distance.

In this study, the angle-based distance matrix, which is a measure of the angle from the starting location of the back trajectories (i.e., Weizhou Island in this study), was used to determine the similarity of different trajectories. The cluster analysis was conducted with the openair R package (version 2.7-1) [31].

The angle-based distance between two trajectories is defined as:

$$d_{1,2}\;=\frac{1}{n}\overset{n}{\underset{i=1}{\sum }}\cos^{-1}\left(0.5\frac{A_{i}+B_{i}+C_{i}}{\sqrt{A_{i}B_{i}}}\right)$$

where

$$A_{i}\;={\left(X_{1}\left(i\right)-X_{0}\right)}^{2}+{\left(Y_{1}\left(i\right)-Y_{0}\right)}^{2}$$

$$B_{i}\;={\left(X_{2}\left(i\right)-X_{0}\right)}^{2}+{\left(Y_{2}\left(i\right)-Y_{0}\right)}^{2}$$

$$C_{i}\;={\left(X_{2}\left(i\right)-X_{1}\left(i\right)\right)}^{2}+{\left(Y_{2}\left(i\right)-Y_{1}\left(i\right)\right)}^{2}$$

where *X*_1_, *Y*_1_ and *X*_2_, *Y*_2_ are the latitude and longitude coordinates of back trajectories 1 and 2, respectively. n is the number of trajectory points. *X*_0_ and *Y*_0_ are the coordinates of the starting location of the back trajectories (i.e., Weizhou Island in this study).

### 2.5. Toxicity Risk Assessment

The toxicity factors and equations used for the data calculation are presented in detail as follows:

The BaP-equivalent carcinogenic power (BaPE) for the total PAHs [32]:


BaPE = BaA × 0.06 + BbF × 0.07 + BkF × 0.07 + BaP × 1.00 + DBA × 0.60 + BPE × 0.08

The total toxicity potency for PAHs relative to 2,3,7,8-tetrachlorodibenzo-*p*-dioxin (TCDD) induction in vitro assays (TEQ) [33]:

$$\mathrm{TEQ}\;=\overset{n}{\underset{i=1}{\sum }}Conc_{i}\times IConc_{i}\;\left(\mathrm{ng}/m^{3}\right)$$

where the average TCDD-IEFs (induction equivalency factors) for the different components studied are listed in Table 2.

The indirect-acting mutagenicities (IDMs) of PAH components, relative to in vitro assays (S. typhimurium TA100 strain with S9 mix) [34], is calculated by:

$$\mathrm{Indirect}-\mathrm{acting}\;\mathrm{mutagenicities}=\overset{n}{\underset{i=1}{\sum }}Conc_{i}\times IDM_{i}\;\left(\mathrm{revertant}/m^{3}\right)$$

where IDMs for the different components studied are listed in Table 2.

## 3. Results and Discussion

### 3.1. Concentrations of PAHs

**Figure 2:**
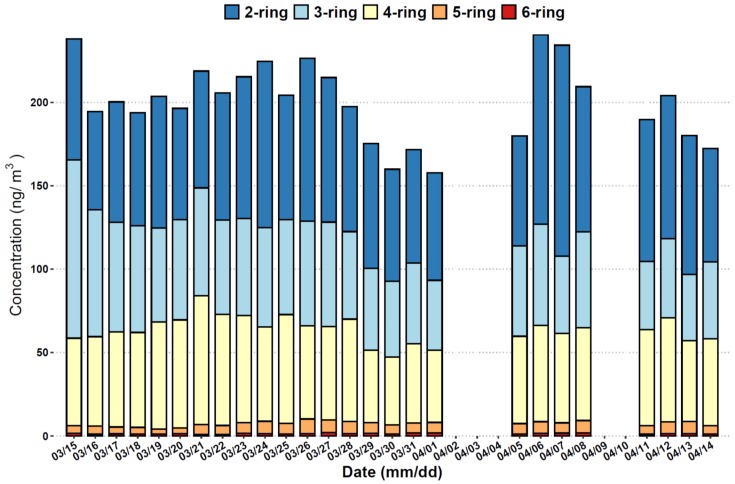
Temporal variations in the concentrations of polycyclic aromatic hydrocarbons (PAHs) with different number of rings during the sampling period.

The temporal variations in the concentrations of PAHs with different number of rings during the sampling period are presented in Figure 2. Samples were not collected on several days (2nd, 3rd, 4th, 9th, and 10th April) due to the intermittent power supply on the island. The total PAHs concentrations tended to increase from 16th March until a periodic high value (226.44 ng/m^3^) was shown on 26th March, followed by a gradual decrease. The highest value of total PAHs concentration (240.71 ng/m^3^) was found on 6th April. The concentration of total 18 PAHs at Weizhou Island ranged from 157.70 to 240.71 ng/m^3^, with a mean of 200.39 ng/m^3^. PAHs with 2 to 4 rings and molecular weights ranging from 128 to 252 contributed dominantly to the total PAHs in each day during the sampling period. The percentages of these PAHs (with 2 to 4 rings) in the total PAHs were from 94.83% to 97.98% in this study.

In Table 3, PAHs concentrations at Weizhou Island were compared with the results from other remote or urban sites in Asia. The PAH levels observed in this study are similar to those at a site in Tibetan Plateau [35], but they are much higher than those of the other listed Asian remote sites in the table, indicating the serious pollution situation in GT. On the other hand, PAH levels at Weizhou Island are still lower than those of some Asian urban sites, such as Beijing, Guangzhou, and Shenyang [6,36,37].

**Figure 3:**
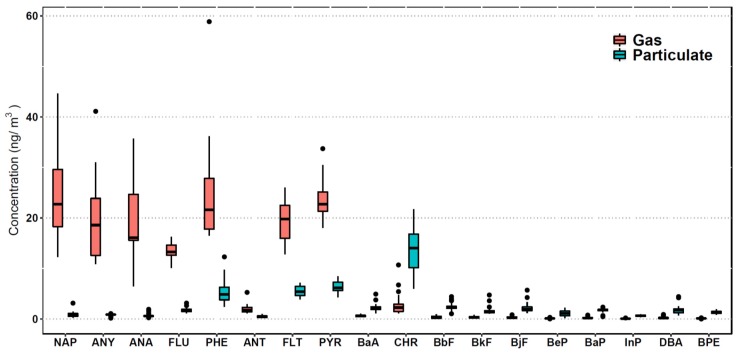
Concentrations of PAHs in the particulate and gas phases.

The average individual PAHs concentrations in the gas and particulate phases during the sampling period are shown in Figure 3. 2-ring PAHs which have low molecular weight were found almost only in the gas phase, whereas the high molecular weight PAHs (5- and 6-ring) were primarily found in the particulate phase. Most of the middle molecular weight PAHs (3- and 4-ring) were distributed in both the gas and particulate phases. Total average concentration of gaseous PAHs, 150.14 ng/m^3^, was 3 times higher than that of particulate PAHs. The above distributions were due to the various subcooled liquid vapor pressures with different molecular weights, and similar distributions were also reported previously [41,42,43,44]. PHE, FLT, and PYR, mainly found in diesel vehicle emissions [45], showed a relatively high concentrations in both the gas and particulate phases in this study. Additionally, the concentration of CHR was also extraordinarily high, especially in the particulate phase. CHR was reported to exhibit high emission factors from both gasoline and diesel vehicles [46,47,48].

### 3.2. Diagnostic Sources of PAHs

**Figure 4:**
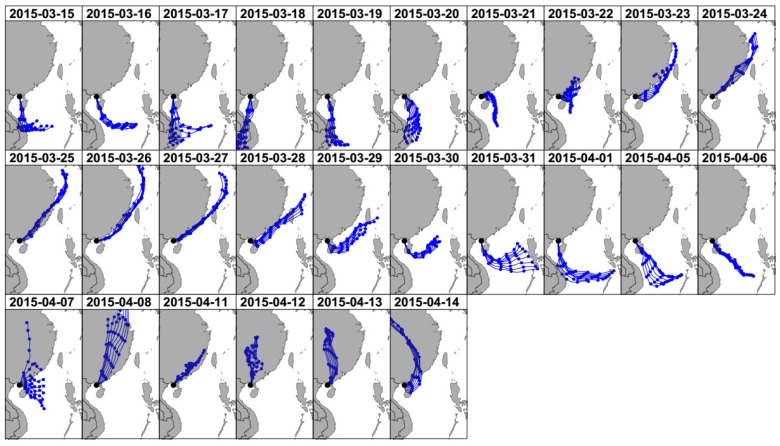
72-h HYSPLIT back trajectories centered Weizhou Island from 15th March to 14th April for each day.

**Figure 5:**
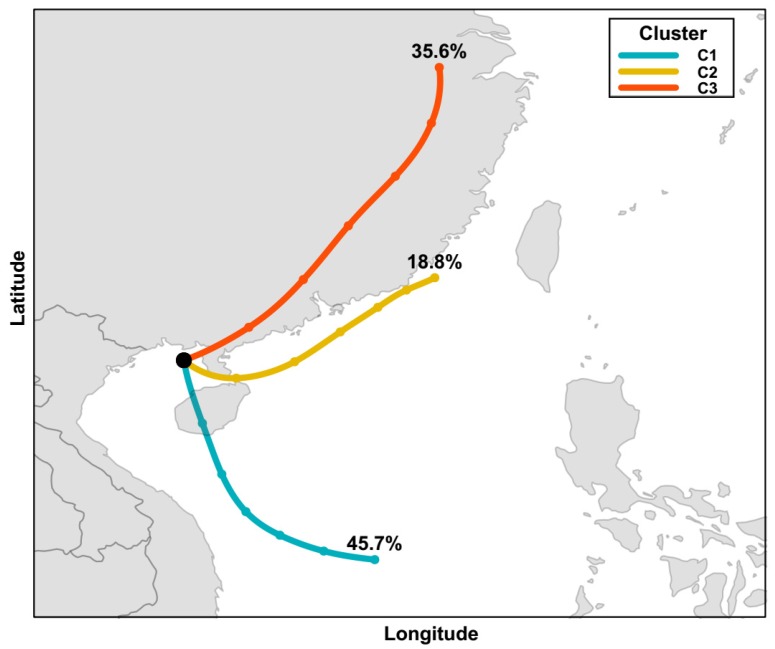
The 3-cluster solution to back trajectories calculated for the Weizhou Island from 15th March to 14th April.

It could be illustrated from Figure 4 that the trajectories were relatively concentrated for each day during the sampling period, and generally originated from a couple of source regions. As shown in Figure 5, three different source regions were identified during this period according to the back trajectories from HYSPLIT model: (1) cluster C1 with southeast orientation, which the air mass may have originated from the Philippines, the South China Sea and the Indo-China Peninsula; (2) cluster C2 with east orientation, which the air mass originated from the remote Yangtze River Delta area, and the trajectories nearly just followed the southeast coastline of China mainland; (3) cluster C3 with northeast orientation, which the air mass originated from China mainland. The proportions of the three clusters were 45.7%, 18.8%, and 35.6%, respectively. The source regions of these three clusters were supposed to have different influences on the concentrations of PAHs at Weizhou Island due to their different emission characteristics. The air mass with trajectories from southeast (cluster C1), east (cluster C2) and northeast (cluster C3) might be affected by spring biomass burning in the northern Southeast Asia, vessel emission from maritime transport, and industrial coal combustion, respectively [49,50,51,52]. For the 26 sampling days, back trajectories were divided into the above three types. The number of sampling days for each type were cluster C1 (southeast, 12 days), cluster C2 (east, 5 days) and cluster C3 (northeast, 9 days).

**Figure 6:**
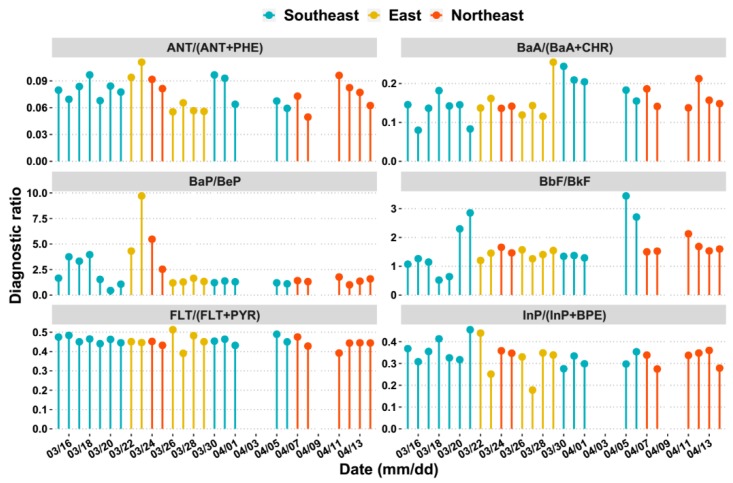
Time series of diagnostic ratio values during sampling period.

As the emission profiles of PAHs congeners strongly depend on their formation mechanisms during the production process of different PAHs such as diesel and gasoline combustion, crude oil processing and coal combustion, diagnostic ratios are frequently used to distinguish different emission sources of PAHs [52]. In this study, the ratios of ANT/(ANT+PHE), BaA/(BaA+CHR), BaP/BeP, BbF/BkF, FLT/(FLT+PYR) and InP/(InP+BPE) were chosen and calculated to diagnose the possible source of PAHs at Weizhou Island, as shown in Figure 6. The ANT/(ANT+PHE) ratio < 0.1 is usually taken as an indicator of petroleum source while a ratio >0.1 indicates a pyrogenic source [52,53]. In this study, the ANT/(ANT+PHE) ratio ranged from 0.05 to 0.11, which implied that the PAHs at the sampling site mainly came from petroleum combustion. The BaA/(BaA+CHR) ratio <0.2 implies the petroleum source, from 0.2 to 0.5 indicates either petroleum or combustion, and a ratio >0.35 implies combustion [54]. The BaA/(BaA+CHR) ratio in this study ranged from 0.08 to 0.25, and the mean value was 0.16, suggesting that the PAHs mainly came from petroleum or combustion. The BbF/BkF ratios of all the samples in this study were higher than 0.5, suggesting an important diesel emission [55]. The FLT/(FLT+PYR) ratio in this study ranged from 0.39 to 0.51, and the mean value was 0.45. The literature suggests that the ratios between 0.4 and 0.5 demonstrate liquid fossil fuel (vehicle and crude oil) combustion whereas ratios >0.50 are characteristic of grass, wood or coal combustion [53]. The ratios of InP/(InP+BPE) for all the samples ranged between 0.16 and 0.5, which indicated that petroleum combustion was an important source of PAHs.

Overall, the above diagnostic ratio results indicate that petroleum combustion is the principal source of PAHs at Weizhou Island. Moreover, it could be found that there was no significant difference in the diagnostic ratios of the different cluster of trajectories, which might be partly explained by the BaP/BeP ratio. BaP is photodegraded more rapidly than its isomer BeP, and the ratio BaP/BeP can be used as a marker of ageing and photodegradation of PAHs. In this study, the mean ratio of BaP/BeP was 2.23, meaning that the PAHs at Weizhou Island were quite fresh and principally influenced by local emissions. As shown in Figure 1, Weizhou Island is in the middle of Gulf of Tonkin and surrounded by several big commercial ports as introduced in Section 1. The possible emission caused by coal and biomass burning from relatively remote source regions might be covered up by local high vessel emissions.

### 3.3. Assessment of Toxicity Risks

The toxicity risks caused by PAHs in the ambient air at Weizhou Island were assessed in Table 4. The sum of 7 carcinogenic PAHs (∑PAH_7_ = BaA + CHR + BbF + BkF + BaP + InP + DBA), BaPE and TEQ are usually used to assess the toxicity risks caused by PAHs [32,33,56,57,58,59]. It has been found that PAHs in ambient airborne particulate matter are the main causes of IDM [34]. Therefore, IDMs determined in one of our previous studies are used [6] to assess the toxicity risks caused by PAHs.

As a result, the toxicities represented by ∑PAH_7_, BaPE and TEQ in particulate phase are significantly higher than those in gas phase (5.2 to 7 times). It is because the toxicities of PAH usually rises with the increasing ring numbers, and high ring PAHs are mainly distributed in particulate phase [33,58]. However, the IDM is only 1.7 times higher in particulate phase than in gas phase, as FLT accounted for 36.6% of the total IDMs, 34.81 and 9.9 rev./m^3^ in gas phase and particulate phase, respectively. Therefore, the toxicities of gas phase should not be neglected from the point of view of IDMs due to the high contribution of FLT at Weizhou Island.

## 4. Conclusions

In this study, PAHs in both the gas and particulate phases were sampled and measured at Weizhou Island in GT from 15th March to 14th April, 2015 for the purpose of exploring the environmental level and possible emission sources of PAHs at a remote island in China. Back trajectory cluster analysis was carried out to distinguish the main source regions, and diagnostic ratios, such as the ANT/(ANT+PHE), BaA/(BaA+CHR), BaP/BeP, BbF/BkF, FLT/(FLT+PYR) and InP/(InP+BPE) ratios, were calculated to diagnose the possible source of PAHs. The toxicity risk of PAHs was also analyzed using the indexes of ∑PAH_7_, BaPE, TEQ and IDMs.

From the results, it could be concluded that:

(1) The PAH levels at Weizhou Island were much higher than those of some other remote sites in Asia, implying more serious pollution of PAHs in GT. PHE, FLT, PYR and CHR, which mainly came from diesel vehicle emission, showed a relatively high concentrations in both the gas and particulate phases.

(2) The comprehensive results of back trajectories and diagnostic ratios analysis demonstrated that the main source of PAHs was probably the local vessel emission, which could even outweigh the influence of biomass burning in the northern Southeast Asia and coal combustion from the mainland.

(3) The toxicities represented by ∑PAH_7_, BaPE and TEQ are much higher in particulate phase than in gas phase, however, the toxicities of gas phase should not be neglected from the point of view of IDMs due to the high contribution of FLT.

Thus, the importance of reducing the emission of PAHs from maritime transportation in GT should be highlighted to control the PAHs pollution in that region. Further basic research should be conducted on the emission inventory, characteristics and toxicity risk of PAHs from marine transport, to provide more scientific suggestion on the human health risk management in coastal regions.

## Figures and Tables

**Table 1:** Investigated PAHs.

No.	Compound	Abbreviation	Rings
1	Naphthalene	NAP	2
2	Acenaphthylene	ANY	3
3	Acenaphthene	ANA	3
4	Fluorene	FLU	3
5	Phenanthrene	PHE	3
6	Anthracene	ANT	3
7	Fluoranthene	FLT	4
8	Pyrene	PYR	4
9	Benz(*a*)anthracene	BaA	4
10	Chrysene	CHR	4
11	Benzo(*b*)fluoranthene	BbF	5
12	Benzo(*k*)fluoranthene	BkF	5
13	Benzo(*j*)fluoranthene	BjF	5
14	Benzo(*e*)pyrene	BeP	5
15	Benzo(*a*)pyrene	BaP	5
16	Indeno(*1,2,3-cd*)pyrene	InP	6
17	Dibenz(*a,h*)anthracene	DBA	5
18	Benzo(*g,h,i*)perylene	BPE	6

**Table 2:** The average 2,3,7,8-tetrachlorodibenzo-*p*-dioxin-induction equivalency factors (TCDD-IEFs) and indirect-acting mutagenicities (IDMs) in S. typhimurium TA100 strain with S9 mix (rev./ng) for each PAH.

PAH	TCDD-IEFs	IDMs	PAH	TCDD-IEFs	IDMs
NAP	-	-	CHR	0.0001	-
ANY	-	-	BbF	-	7.8
ANA	-	-	BkF	-	3.8
FLU	-	-	BjF	-	-
PHE	-	-	BeP	-	-
ANT	-	-	BaP	0.0001	16.1
FLT	0.00000001	1.8	InP	-	0.8
PYR	-	-	DBA	0.0001	5.0
BaA	0.00001	2.0	BPE	0.00000001	0.3

**Table 3:** Comparison of the total PAHs (ng/m^3^) at Weizhou Island with some other sites in Asia.

Type	Site	Particulate Phase	Gas Phase	∑PAH Concentrations	Congener No.	References
Remote	Weizhou Island	40.19–61.86	116.22–186.74	157.70–240.71	18	This study
	Tibetan Plateau	4.4–60	79–350	87–360	15	[35]
	Tuoji Island	4.24–40.62	-	-	15	
	Hengchun Peninsula	0.01–1.36	0.39–2.31	0.42–2.79	16	[38]
	Amami Sea and Japan Sea	0.03–2.11	-	-	12	[29]
	Jeju Island	0.404–2.93	-	-	21	[27]
	Gosan	2.9	1.4	-	14	[39]
Urban	Donghe	12.9–348.8	-	303.9–1616.5	16	[36]
	Beijing	3.2–222.7	-	131.0–979.3	16	[36]
	Guangzhou	2.2–90.5	25.7–239.5	27.9–329.4	16	[40]
	Shenyang	-	-	92.6–316(cold season)	9	[37]

**Table 4:** Potential toxicity risk of PAHs.

	Gas Phase	Particulate Phase	Total PAHs
∑PAH_7_	4.65	23.95	28.60
BaPE	0.46	3.35	3.81
TEQ	0.00033	0.00172	0.00206
IDMs	44.71	77.37	122.08
